# Phase transformation mechanism of MnCO_3_ as cathode materials for aqueous zinc-ion batteries

**DOI:** 10.3389/fchem.2022.954592

**Published:** 2022-08-05

**Authors:** Junjie Zheng, Pengcheng Liu, Jia Yao, Yi Gan, Jingying Li, Cong Wang, Xiang Liu, Yiheng Rao, Guokun Ma, Lin Lv, Hanbin Wang, Li Tao, Jun Zhang, Hao Wang

**Affiliations:** ^1^ School of Microelectronics and Faculty of Physics and Electronics Science, Hubei University, Wuhan, China; ^2^ Hubei Yangtze Memory Laboratories, Wuhan, China

**Keywords:** MnCO_3_, doping, zinc-ion battery, phase transformation, cyclic stability

## Abstract

Aqueous rechargeable zinc-ion batteries (ZIBs) have been given more and more attention because of their high specific capacity, high safety, and low cost. The reasonable design of Mn-based cathode materials is an effective way to improve the performance of ZIBs. Herein, a square block MnCO_3_ electrode material is synthesized on the surface of carbon cloth by a one-step hydrothermal method. The phase transition of MnCO_3_ was accompanied by the continuous increase of specific capacity, and finally maintained good cycle stability in the charge–discharge process. The maximum specific capacity of MnCO_3_ electrode material can reach 83.62 mAh g^−1^ at 1 A g^−1^. The retention rate of the capacity can reach 85.24% after 1,500 cycles compared with the stable capacity (the capacity is 61.44 mAh g^−1^ under the 270th cycle). *Ex situ* characterization indicates that the initial MnCO_3_ gradually transformed into MnO_2_ accompanied by the embedding and stripping of H^+^ and Zn^2+^ in charge and discharge. When MnCO_3_ is no longer transformed into MnO_2_, the cycle tends to be stable. The phase transformation of MnCO_3_ could provide a new research idea for improving the performance of electrode materials for energy devices.

## 1 Introduction

With the rapid development of information and intelligence in human society and the growth of new energy demand, secondary batteries play an important role in the application of new energy (Deng et al., 2021; Zhu et al., 2021). Lithium-ion batteries were developed for market application because of their excellent energy density, power density, and cycle life (Wang C et al., 2020; Hou et al., 2021; Hua et al., 2021). However, the further development of lithium-ion batteries is seriously restricted and challenged by their lack of resources, high cost, and potential safety hazards of organic electrolytes (Gan et al., 2020; Cai et al., 2021; Shan et al., 2021; Wang et al., 2021; Cui et al., 2022). The supplementary scheme of lithium-ion batteries has gradually become an urgent problem.

Aqueous zinc-ion batteries stand out among many electrochemical energy-storage devices because of their outstanding advantages such as safety, low cost, high-energy density, and environmental friendliness (Wang T et al., 2020; Du et al., 2020; Zhou and Guo, 2021). At present, the common cathode materials are V-based materials (Li J et al., 2020; Zhang L et al., 2020; Deng et al., 2020; Zhang et al., 2021), Mn-based materials (Wang J et al., 2020; Tan et al., 2020; Mao et al., 2021), and Prussian blue (Li Z et al., 2020). Among them, Mn-based oxides are widely used as cathode materials for aqueous zinc-ion batteries due to their low cost and abundant crystal structures (MnO, MnO_2_, Mn_2_O_3_, Mn_3_O_4_, etc.) (Zhao et al., 2019a; Gao et al., 2020). In 2012, Xu proposed a safe and environmentally friendly battery, which was made with α-MnO_2_ as cathode, zinc plate as anode, and ZnSO_4_ aqueous as electrolyte. It is first indicated that the Zn^2+^ intercalation and desorption mechanism based on Zn^2+^ was inserted into α-MnO_2_ (Xu et al., 2012). Ji et al. reported a multi-valence cobalt-doped Mn_3_O_4_ with high capacity (Ji et al., 2020). Zhu et al. reported the activation of MnO by inducing Mn defects, wherein the Mn defects are formed through a charging process that converts the MnO with poor electrochemical activities toward Zn^2+^ into high electrochemically active cathode for aqueous ZIBs (Zhu C et al., 2020). However, the diversity of valence states of Mn-based oxides will lead to more side reactions, resulting in irreversible phase transition in the reaction process. It will damage the cycle stability and capacity. Thus, the new material system needs to be further excavated.

In recent years, the crystal structure of MnCO_3_ is similar to that of MnO_2_ composed of [MnO_6_] and CO_3_^2−^; generally, MnO_2_ is synthesized through the decomposition of Mn-based oxyacid salt and hydroxide. However, this process is more complicated and energy consuming than that of MnCO_3_. Consequently, MnCO_3_ has been gradually used in electrochemical energy-storage devices due to its rich reserves, environmental friendliness, and simple synthesis (Zhong et al., 2015; Li et al., 2018). It is a potential high-performance anode material for electrochemical energy-storage devices (Yao et al., 2021). Yao et al. reported an electrodeposited MnCO_3_ as a high-performance electrode material for supercapacitors (Yao et al., 2021). Liu et al. reported *in situ* N-doped MnCO_3_ anode material via one-step solvothermal for lithium-ion batteries (LIBs) (Liu et al., 2020). Zhao et al. reported that when MnCO_3_–RGO composite anode materials are used as anode material, they deliver a large capacity of 873 mAh g^−1^ even after 400 cycles at 1°C (Zhao et al., 2019b). However, their chemical properties are more active, and charge transfer occurs in the process of phase transition, which provides a better cycle stability. At present, the application of MnCO_3_ for ZIBs is still very rare, and its zinc storage mechanism has not been deeply explored.

Herein, we synthesized a square block MnCO_3_ electrode material on the surface of carbon cloth by the one-step hydrothermal method. The phase transition of MnCO_3_ was accompanied by the continuous increase of specific capacity and finally maintained good cycle stability in the charge–discharge process. The maximum specific capacity of MnCO_3_ electrode material can reach 83.62 mAh g^−1^ at 1 A g^−1^. The retention rate of the capacity can reach 85.24% after 1,500 cycles compared with the stable capacity (the capacity is 61.44 mAh g^−1^ under the 270th cycle). *Ex situ* characterization indicates that the initial MnCO_3_ gradually transformed into MnO_2_ accompanied by the embedding and stripping of H^+^ and Zn^2+^ in charge and discharge. When MnCO_3_ is no longer transformed into MnO_2_, the cycle tends to be stable. The phase transformation of MnCO_3_ could provide a new research idea for improving the performance of electrode materials for energy devices.

## 2 Experimental section

### 2.1 The synthesis of MnCO_3_/CC

The prepared empty carbon cloth (CC) was immersed in a beaker containing concentrated nitric acid and heated in a water bath at 80°C for 2–3 h, then repeatedly washed with deionized water and absolute ethanol, and finally dried for future use. After pretreatment of empty CC, 10 mmol Mn(CH_3_COO)_2_, 50 mmol urea, and 40 ml of deionized water were added to 100 ml of Teflon lining. After stirring for 5 min, putting a 3 cm*2 cm carbon cloth, the mixed solution was placed in an autoclave and kept at 100°C for 16 h. The carbon cloth after the reaction was washed three times with deionized water and ethanol, and the sample (MnCO_3_/CC) was obtained after drying at 80°C for one night.

### 2.2 Materials characterization

The crystallographic features of the as-prepared samples were characterized by X-ray diffraction (XRD, Philips X'Pert PRO, Cu Kα, λ = 0.1542 nm). The morphology and detailed microstructure were conducted on a scanning electron microscope (SEM, FEI Quanta 200) and transmission electron microscopy (TEM, Philips, Tecnai G220), coupled with energy-dispersive X-ray spectroscopy (EDS). The element composition and surface chemical state were recorded on an X-ray photoelectron spectroscope (XPS, Kratos AXIS Ultra DLD-600W).

### 2.3 ZIBs performance test

In this experiment, the cathode material was MnCO_3_ grown *in situ* with carbon cloth, and the anode material was selected from commercial zinc foils with high purity. The electrolyte was 2 M zinc sulfate, and 0.2 M manganese sulfate was added as the electrolyte compensation. The two electrodes were separated by a glass fiber separator. Cyclic voltammetry (CV) and galvanostatic charge–discharge (GCD) were performed on the ChenHua electrochemical workstation (CHI760E) to test the electrochemical performance and used the eight-channel battery test equipment (NEWARE) to test rate performance and cycle life.

## 3 Results and discussion

### 3.1 Structure characterization of MnCO_3_/CC

**FIGURE 1:**
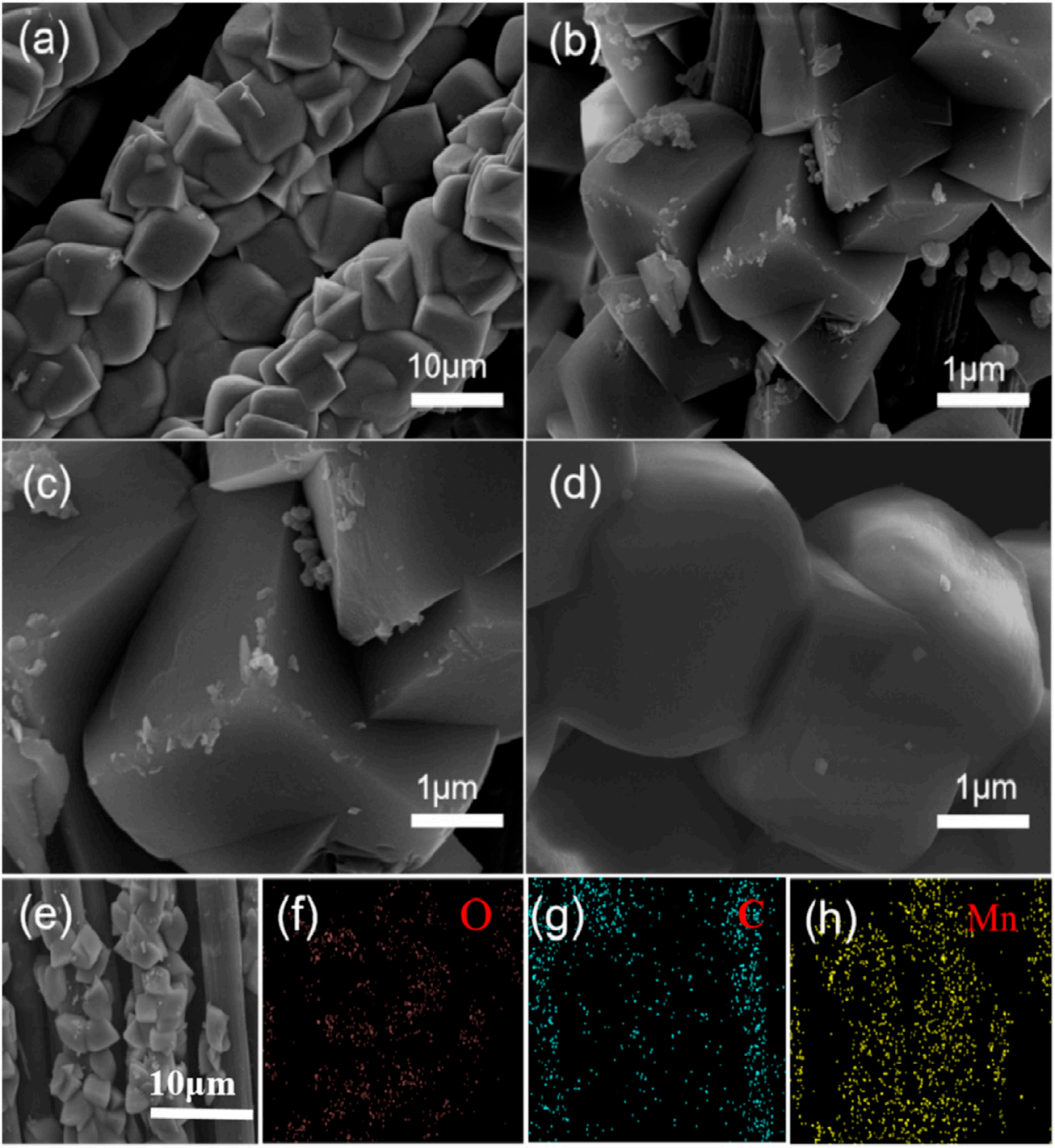
**(A–D)** SEM images of MnCO_3_@CC **(E–H)** EDS images of MnCO_3_@CC.

**FIGURE 2:**
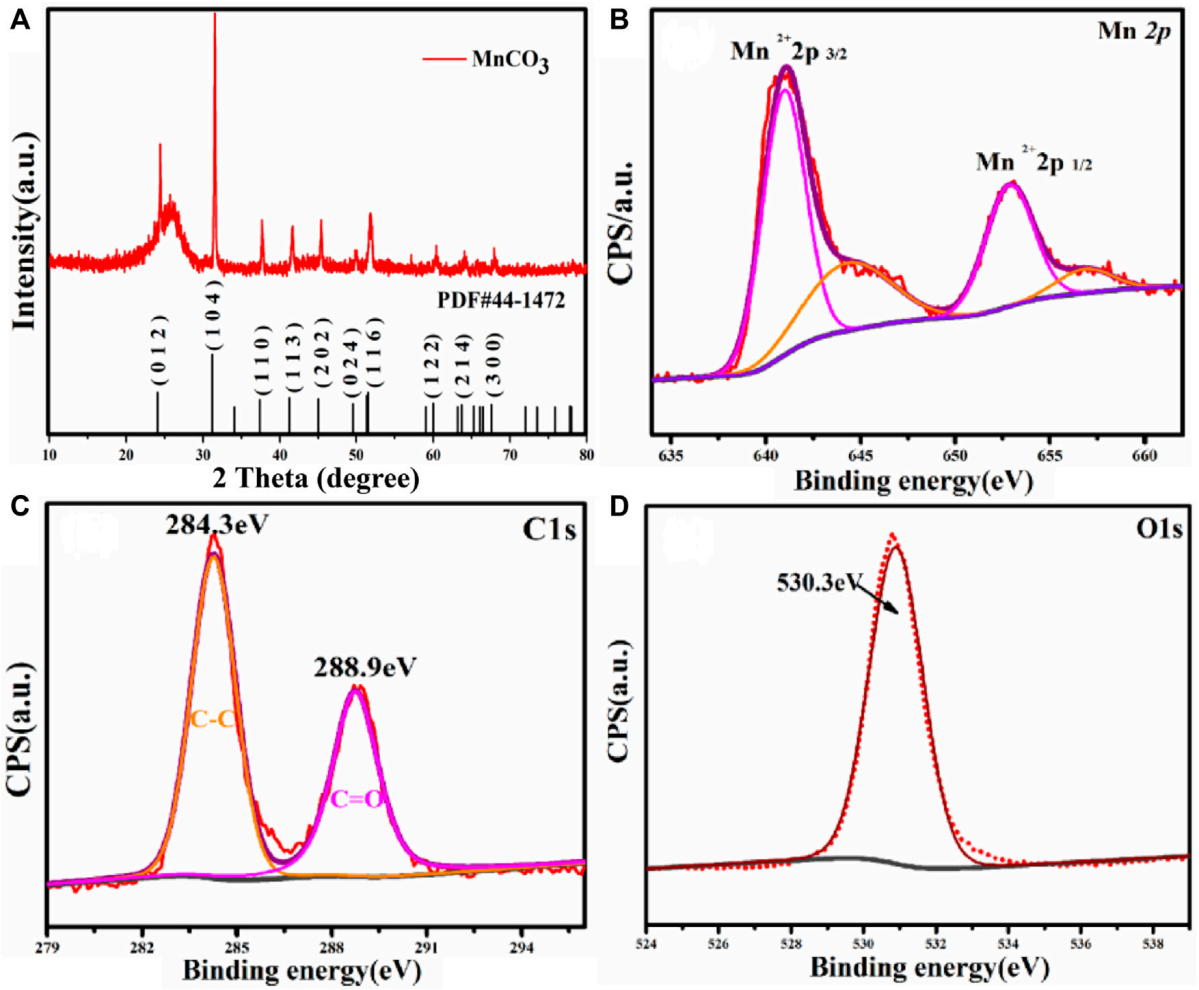
**(A)** XRD pattern of MnCO_3_@CC. XPS pattern of MnCO_3_@CC: **(B)** Mn2p, **(C)** C1s, and **(D)** O1s.

The micro-morphology of MnCO_3_@CC was characterized by a scanning electron microscope (SEM), as shown in Figure 1. Figures 1A–D show that the as-synthesized MnCO_3_ cubes are grown evenly on the carbon cloth, and the surface of MnCO_3_ is relatively smooth. Figures 1F–H show the EDS element distribution diagram of C, Mn, and O, respectively. The results are basically consistent with the MnCO_3_. The crystal structure of the sample was analyzed by XRD, as shown in Figure 2A. The diffraction peaks can be well indexed to the representative peaks of the MnCO_3_ phase (JCPDS 44-1,472) (Zhu L et al., 2020; Zhu J et al., 2020). In detail, the peaks at 24.25°, 31.36°, 37.52°, 41.42°, 45.18°, 49.67°, 51.68°, 60.13°, 63.88°, and 67.70° correspond to (012), (104), (110), (113), (202), (024), (116), (122), (214), and (300) crystal planes of MnCO_3_, respectively. In addition, there are no other heteropeaks in the XRD spectrum, which prove that the material we synthesized is pure MnCO_3_ cathode material. Previously, EDS and XRD confirmed that there were three elements, Mn, C, and O, in the cathode material synthesized by the hydrothermal method in the experimental process, and the phase accorded with the diffraction results of MnCO_3_.

In order to further confirm the structure of the samples, the valence states of the synthetic materials were analyzed by XPS. Figure 2B demonstrates two typical Mn2p1/2 and Mn2p3/2 orbitals of the MnCO_3_ phase. The binding energies of the two main peaks are 641.02 and 653.04 eV, with a difference of 12.02 eV, indicating that the valence of Mn in the compound is +2 (Zhang B et al., 2020). In the C1s spectrum in Figure 2C, the binding energies of the two main peaks are 284.3 and 288.9 eV, corresponding to C–C and C = O. In Figure 2D, the binding energy corresponding to the O1s is 530.3 eV, which further illustrates that O and C are combined and are expressed in the form of CO_3_^2−^ (Wang N et al., 2020). Supplementary Figure S1 shows the representative XPS survey spectrum of the sample, which verifies the presence of Mn, O, and C elements, indicating that the sample synthesized in the experiment is MnCO_3_ without other impurities. This conclusion is consistent with the XRD results in Figure 2A.

**FIGURE 3:**
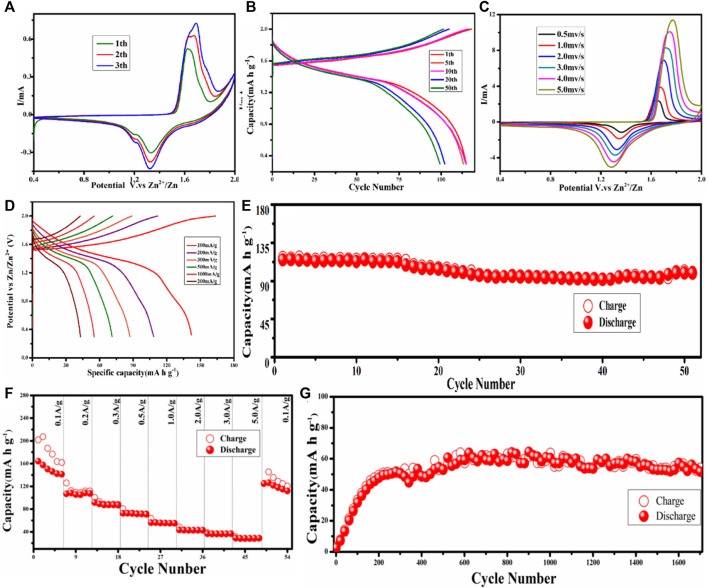
**(A)** CV curves of the first three cycles of MnCO_3_ at 0.5 mV/s. **(B)** Galvanostatic charge–discharge (GCD) curves under different cycles. **(C)** CV curves of MnCO_3_ at different scan rates (0.5–5 mV/s). **(D)** GCD curves under different current densities. **(E)** The cycle performance of MnCO_3_ at 0.2 A g^−1^. **(F)** Rate performance of MnCO_3_. **(G)** The cycle performance of MnCO_3_ at 2.0 A g^−1^.

The electrochemical performance of MnCO_3_@CC cathode material is carried out in the coin cell, which utilized zinc foil as the anode and 2 M ZnSO_4_ + 0.2 M MnSO_4_ as the electrolyte. Cyclic voltammetry (CV) curves of unactivated MnCO_3_@CC are obtained at the scan rate of 0.5 mV/s under 0.4–2.0 V, as shown in Figure 3A. In the first cycle, the oxidation potential is 1.63 V, corresponding to two reduction potentials of 1.20 and 1.33 V, respectively. In the second cycle, the original oxidation potential shifted to the left to 1.64 V, while the second oxidation potential appeared at 1.69 V and the reduction potential remained at 1.20 and 1.33 V, respectively. There are also two oxidation potentials in the third circle, which are 1.65 and 1.69 V, respectively. The reduction potential is consistent with the first two cycles, and their response current and peak intensity further increase. The change of oxidation potential should be caused by the phase transition process of Mn ions, corresponding to Mn^3+^ to Mn^4+^, indicating that there is a gradual activation process in the initial stage. The two reduction peaks are mainly attributed to the embedding and stripping behavior of H^+^ and Zn^2+^. Galvanostatic charge–discharge (GCD) curves under different cycles at 0.1 A g^−1^ are shown in Figure 3B. There is only one discharge platform in 1.33 V under 1, 5, 10, 20, and 50 cycles, which is due to the embedding behavior of Zn^2+^ in the reaction process after the cathode material reaches a stable phase. Figure 3C shows the CV curves of MnCO_3_@CC at different scanning rates from 0.5 mV/s to 5 mV/s after activation. It is indicated that the response current gradually increases with the increase of the scanning rate. Figure 3D shows GCD curves under different current densities. The specific capacity is the highest at 0.1 A g^−1^, but the Coulomb efficiency is the lowest because the phase change is the most obvious at this stage, which is basically consistent with the previous conclusions.

After the first cycle, Figure 3E shows the cyclic test of MnCO_3_ at 0.2 A g^−1^. Because the cathode material reaches a stable state after the activation process, its capacity remains stable during the following cycling. The first cycle capacity of the cathode material is 114.82 mAh g^−1^, the cycle capacity of the 50th cycle is 99.26 mAh g^−1^, and the retention rate is 87%. Figure 3F shows the rate performances of MnCO_3_ at 0.1, 0.2, 0.3, 0.5, 1.0, 2.0, 3.0, and 5.0 A g^−1^ in 0.4–2.0 V. The specific capacities are 164.17, 108.30, 91.52, 72.64, 56.23, 43.24, 36.59, and 28.89 mAh g^−1^, respectively. At low current density, the capacity of MnCO_3_@CC begins to remain stable after reaching a stable phase. In the subsequent current density, its capacity remains basically stable, indicating that the MnCO_3_@CC has good stability and rate performance under different current densities. Figure 3G shows the cycle performance of MnCO_3_/CC at 2.0 A g^−1^. At the higher current density, the cycle curve also rises slowly at first and then remains stable. The initial capacity of the MnCO_3_@CC is also low. After 400 cycles, the capacity reaches 48.46 mAh g^−1^ and then remains stable. After 1,700 cycles, when the capacity is relatively stable, the retention rate can reach 112.5% compared to the 400^th^ cycle, which proves the good stability of the material.

**FIGURE 4:**
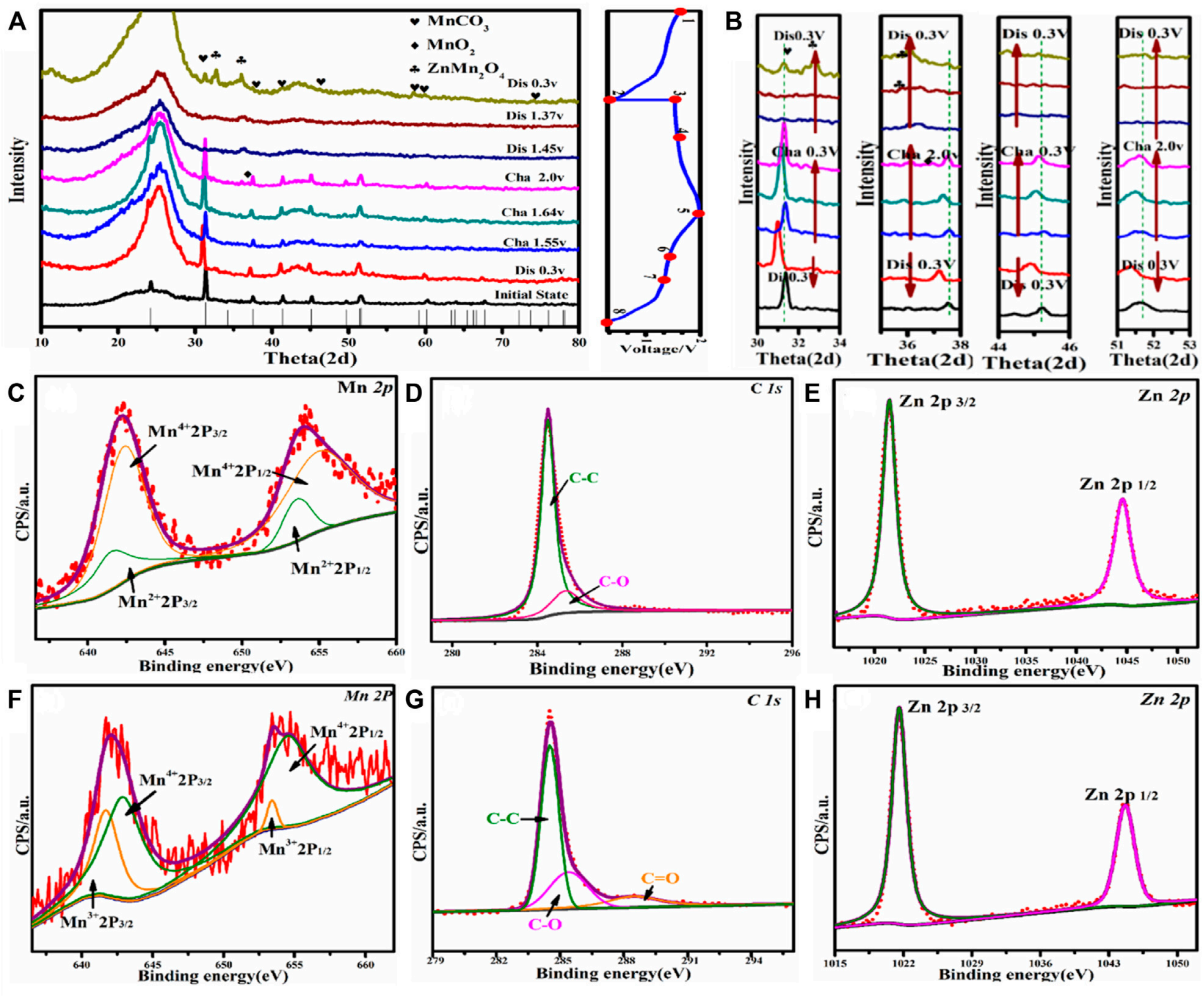
**(A)** EX-XRD patterns of MnCO_3_@CC at 0.1 A g^−1^. **(B)** Angular shifts of different diffraction peaks. **(C–E)** The XPS patterns of MnCO_3_@CC for Mn, C, and Zn elements under charge to 2.0 V at 0.1 A g^−1^. **(F–H)** The XPS patterns of MnCO_3_@CC for Mn, C, and Zn elements under discharge to 0.3 V at 0.1 A g^−1^.

To further explore the phase change and ion intercalation mechanisms of MnCO_3_@CC cathode material, the electrodes were tested by EX-XRD, SEM, and XPS in different stages of charge and discharge. Figure 4 shows the EX-XRD patterns of nanocube MnCO_3_@CC at 0.1 A g^−1^. In the initial (position 1), only the original peak of MnCO_3_ exists on the electrode (Figure 4A). When charging to 1.68 and 2.0 V, the peak of MnO_2_ appears near 36.8°, but its peak intensity is weak, indicating that little amount of MnCO_3_ changes into MnO_2_ during charging. In the discharge, no strong peak of MnCO_3_ is observed at positions 6, 7, and 8 in Figure 4A, but the MnO_2_ diffraction peak is obvious near 36.8°, which indicated that MnO_2_ is generated by the phase transformation of some MnCO_3_. When discharging to 0.3 V, the results show that the ZnMn_2_O_4_ phase appears near 32.9° and 38.9°, indicating that Zn^2+^ embedding behavior occurs in this process. In the charging process (Figure 4B), the diffraction peak deviates to the left between 30° and 34°, 36° and 38°, 44° and 46°, and 51° and 53°, and the crystal plane spacing becomes smaller, which is affected by the stripping behavior of Zn^2+^. Supplementary Figure S2 shows the SEM images of the electrode charging to 2.0 V and discharging to 0.3 V at 0.1 A g^−1^. Supplementary Figures S2A–S2C are the SEM images of charging to 2.0 V. After the original reaction, the smooth surface of MnCO_3_ is gradually rough, and the cube is stacked by sheets. Supplementary Figures S2D–S2E are the SEM images after discharging to 0.3 V. The results show that there are lines on the surface of cube edges and corners, which increases its specific surface area. It is conducive to the ion transmission between the host and Zn^2+^ and improves the electrochemistry performance of the cathode material.

Figures 4C–E show the Mn, C, and Zn XPS spectrums of MnCO3@CC cathode material under charging to 2.0 V at 0.1 A g^−1^. Figure 4C shows the high-resolution Mn2p spectrum. These two groups of spin-orbit resolution peaks can be decomposed into Mn^2+^ (2p3/2, 641.4 eV; 2p1/2, 653.2 eV) and Mn^4+^ (2p3/2, 643.2 eV; 2p1/2, 655.6 eV). The content of Mn^2+^ is much greater than that of Mn^4+^ because only a small amount of MnCO_3_ is transformed into MnO_2_ in the charging stage, and a large number of cathode materials have not been activated, resulting in two valence states of +2 and +4 at the same time, and the proportion difference is large. The high-resolution spectrum of element C shown in Figure 4D is consistent with the reaction process. Figure 4E shows the high-resolution spectrum of Zn. The existence of Zn^2+^ (2p3/2, 1,044.5 eV; 2p1/2, 1,021.4 eV) is caused by the residue of ZnSO_4_ in the electrolyte.

The sample was discharged to 0.3 V for XPS analysis at 1.0 A g^−1^. In the high-resolution spectrum of Mn2p shown in Figure 4F, the two groups of spin orbits can be decomposed into Mn^3+^(2p3/2, 642.4 eV; 2p1/2, 654.2 eV) and Mn^4+^ (2p3/2, 644.1 eV; 2p1/2, 655.6 eV). The existence of Mn^3+^ and Mn^4+^ further proved the formation of MnO_2_ during charging and the embedding of Zn^2+^ in MnO_2_ during discharge, and ZnMn_2_O_4_ with spinel structure was formed. Due to the low content of MnO_2_ generated in the charging stage and since only part of MnO_2_ reacts with Zn^2+^ in the discharge process, the content of Mn^3+^ should be much less than Mn^4+^. Mn^2+^ was not detected because the generated MnO_2_ covered the original material, so its valence state could not be detected. Figure 4H shows the high-resolution spectrum of Zn2p. Zn is +2 valence in the test sample, which is consistent with the embedding behavior of Zn^2+^.

**FIGURE 5:**
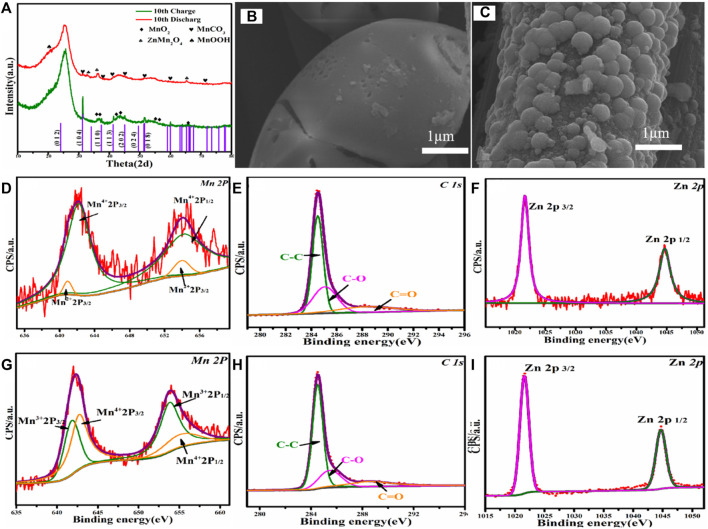
**(A)** The XRD pattern of MnCO_3_@CC in charge to 2.0 V and discharge to 0.3 V at 0.1 A g^−1^ after 10 cycles. **(B)** The SEM image of MnCO_3_@CC under charge to 2.0 V at 0.1 A g^−1^ after 10 cycles. **(C)**The SEM image of MnCO_3_@CC under discharge to 0.3 V at 0.1 A g^−1^ after 10 cycles. **(D–F)** The XPS patterns of MnCO_3_@CC for Mn, C, and Zn elements under charge to 2.0 V at 0.1 A g^−1^ after 10 cycles. **(G–I)** The XPS patterns of MnCO_3_@CC for Mn, C, and Zn elements under discharge to 0.3 V at 0.1 A g^−1^ after 10 cycles.

In order to further explore the activation mechanism and ion intercalation/desorption behavior of the MnCO_3_/CC, the batteries were analyzed after 10 cycles at a low current density of 0.1 A g^−1^. Figure 5A shows the EX-XRD patterns of charging to 2.0 V and discharging to 0.3 V after 10 cycles. After charging, the original MnCO_3_ diffraction peak gradually disappears, while the MnO_2_ characteristic peak becomes more and the peak intensity becomes stronger. In the discharge stage, a large amount of Zn^2+^ is embedded in MnO_2_ rather than MnCO_3_ and transformed into the spinel ZnMn_2_O_4_. The diffraction peak of ZnMn_2_O_4_ is positively correlated with MnO_2_ content, which is also the reason for the continuous increase in the electrochemical capacity of cathode materials. When MnCO_3_ is not in a phase transition to MnO_2_, the cycle curve reaches stability. Figure 5B shows the SEM image of the MnCO_3_@CC charged to 2.0 V after 10 cycles. The initial MnCO_3_@CC smooth cube block edges and corners basically disappear, and cracks appear on the surface. The change of morphology is conducive to expand the contact area between Zn^2+^ and the host, reduce the ion and charge transmission path and resistance, and then improve performance. Figure 5C shows the SEM image of the MnCO_3_@CC discharged to 0.3 V after 10 cycles. The result shows that the carbon cloth is covered by nano-spheres, the original nanocube basically disappears, and the specific surface area continues to increase.

The gradual change of morphology is also the reason for the continuous increase of specific capacity. The XPS patterns of Mn are shown in Figure 5D under charging to 2.0 V after 10 cycles. The two groups of spin orbits can be decomposed into Mn^2+^ (2p3/2, 641.5 eV; 2p1/2, 653.8 eV) and Mn^4+^ (2p3/2, 641.5 eV; 2p1/2, 653.8 eV), and the contents of Mn^2+^ and Mn^4+^ are opposite to the results after the first cycle. At this time, the proportion of Mn^4+^ is much larger than Mn^2+^, which further proves that MnCO_3_ gradually changes into MnO_2_. The high-resolution spectrum of Mn2p is shown in Figure 5H, the two groups of spin orbits can be decomposed into Mn^3+^ (2p3/2, 642.3 eV; 2p1/2, 654.2 eV) and Mn^4+^ (2p3/2, 644.2 eV; 2p1/2, 655.8 eV). After long-time activation, a large number of MnCO_3_ change into MnO_2_, and the increase of MnO_2_ gradually increases the content of discharge product ZnMn_2_O_4_. Therefore, the proportion of Mn^3+^ and Mn^4+^ is basically the same. The changes in valence and content further prove the mechanism that the MnCO_3_ first changes into MnO_2_, and then the generated MnO_2_ reacts with Zn^2+^ in the electrolyte.

## 4 Conclusion

The MnCO_3_ nanocubes are synthesized on carbon cloth by the one-step hydrothermal method, which has good electrochemical performance as cathode material for ZIBs. The specific capacity of the MnCO_3_/CC shows 82.73 mAh g^−1^ at 1.0 A g^−1^ after long-time activation. After 1,500 cycles, the capacity retention rate is 110.6% compared with that at 200 cycles, which indicates that the MnCO_3_@CC has excellent stability in the charging and discharging process. The Zn^2+^ storage mechanism of the MnCO_3_@CC was explored by *in situ* SEM, XRD, and XPS. In the initial stage, MnCO_3_ transformed into MnO_2_, and the generated MnO_2_ reacted with Zn^2+^ in the electrolyte. In the discharge stage, the spinel ZnMn_2_O_4_ is gradually formed in the cathode with the embedding of Zn^2+^. In the charging stage, the ZnMn_2_O_4_ is gradually transformed into MnO_2_ with the removal of Zn^2+^. In this study, the MnCO_3_ cathode material can achieve high specific capacity and cycle stability, which provides a new idea for high-performance aqueous zinc-ion batteries.

## Data Availability

The original contributions presented in the study are included in the article/Supplementary Material; further inquiries can be directed to the corresponding authors.
