# Supplementary material for: Phase transformation mechanism of MnCO3 as cathode materials for aqueous zinc-ion batteries
Source: Front Chem. 2022 Aug 5;10:954592. doi: 10.3389/fchem.2022.954592 (PMC9388732; doi:10.3389/fchem.2022.954592)
Supplement: Supplementary file 1 [file DataSheet1.docx]

**Phase transformation mechanism of MnCO_3_ as cathode materials for aqueous zinc ion batteries**

Junjie Zheng^a,b,1^, Pengcheng Liu^a,b,1^, Jia Yao^a^, Yi Gan^a^, Jingying Li^a^, Cong Wang^a^, Xiang Liu ^a^, Yiheng Rao^a,b*^, Guokun Ma^a,b^, Lin Lv^a,b^, Hanbin Wang^a,b^, Li Tao^a,b^, Jun Zhang^a,b*^, Hao Wang^a,b^

^a^ School of Microelectronics and Faculty of Physics and Electronics Science, Hubei University, Wuhan 430062, PR China.

^b^ Hubei Yangtze Memory Labs, Wuhan, 430223, China.

*Corresponding author. Fax: +86 27 88663390. E-mail: [yihengrao@163.com (Yiheng Rao);](mailto:yihengrao@163.com;) gwen_zhang@126.com (Jun Zhang)

^1^ These authors have contributed equally to this work.


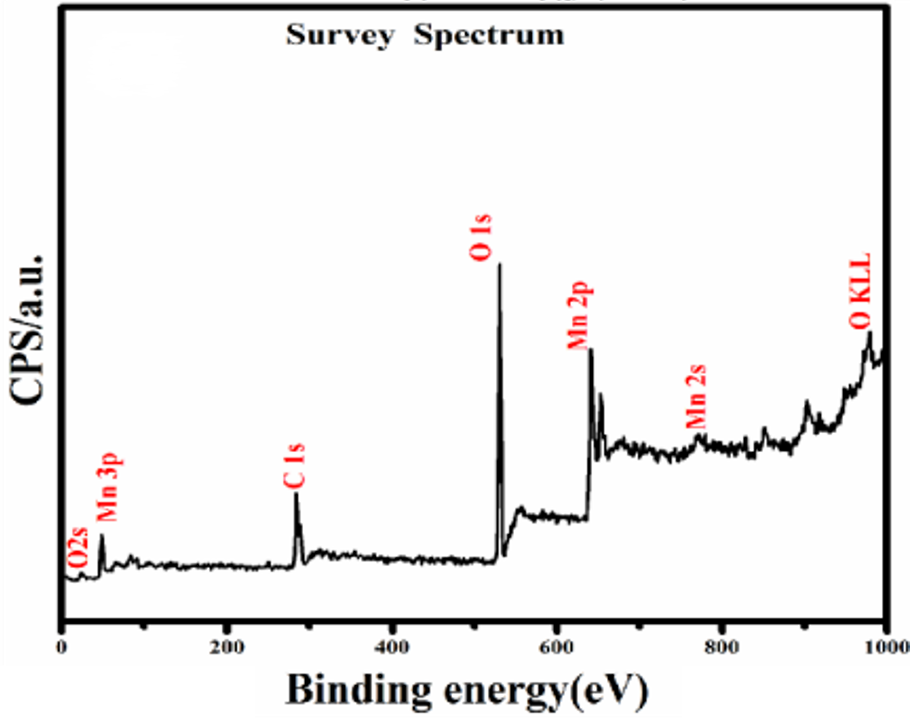


Fig. S1 XPS survey spectrum of the MnCO_3_/CC


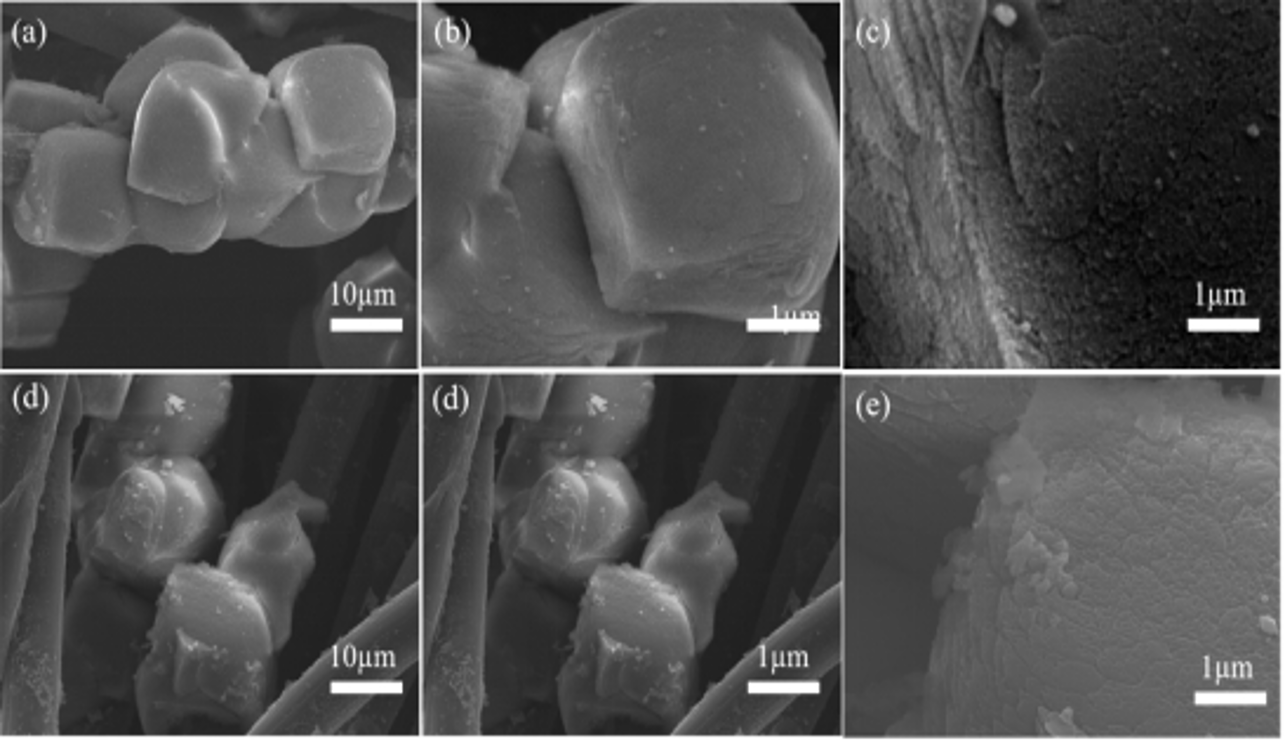


Fig. S2 SEM images of the electrode at charging to 2.0 V and discharging to 0.3 V at 0.1 A g^-1^.
